# High-Definition Liposuction: An Expert Video Roundtable Discussion

**DOI:** 10.1093/asjof/ojae056

**Published:** 2024-07-16

**Authors:** Jeffrey A Gusenoff, David Sieber, Douglas Steinbrech

The authors are pleased to present an expert roundtable discussion on high-definition liposuction (Video). The discussants include a panel of leading experts in aesthetic surgery, and the video roundtable was recorded live at The Aesthetic Meeting 2024 in Vancouver, Canada. The panelists began with an explanation of the differences between traditional liposuction and high-definition liposuction, namely the targeted removal of fat through high-definition liposuction to highlight certain areas of the patient's anatomy, as opposed to the simple removing of excess fat in traditional liposuction. They agreed that patient selection is crucial, as patients with more visceral fat may not be good candidates for high-definition liposuction. The panelists elaborated on new ultrasound technologies that have emerged, touched on common adverse events and how to avoid them, and discussed the use of augmentation and fat transfer to improve and accent results in high-definition liposuction patients. They concluded by discussing their postoperative processes, including the use of drains, compression, and massages, so that patients can adequately recover from surgery and avoid complications.

